# Genomic Characterization of Wild *Lactobacillus delbrueckii* Strains Reveals Low Diversity but Strong Typicity

**DOI:** 10.3390/microorganisms12030512

**Published:** 2024-03-02

**Authors:** Anna Grizon, Sébastien Theil, Sandra Helinck, Pauline Gerber, Pascal Bonnarme, Christophe Chassard

**Affiliations:** 1 UMR 0545 Fromage, INRAE, VetAgro Sup, Université Clermont Auvergne, 20 Côte de Reyne, F-15000 Aurillac, France; anna.grizon@inrae.fr (A.G.); sebastien.theil@inrae.fr (S.T.); 2 UMR SayFood, INRAE, AgroParisTech, Université Paris-Saclay, F-91120 Palaiseau, France; sandra.helinck@inrae.fr; 3 Pôle Fromager AOP Massif Central, F-15000 Aurillac, France; pauline.gerber@pole-fromager-aop-mc.fr

**Keywords:** *Lactobacillus delbrueckii*, intraspecies diversity, autochthonous starter culture, genetic diversity, volatile compounds production

## Abstract

Investigating the diversity of a given species could give clues for the development of autochthonous starter cultures. However, few studies have focused on the intraspecies diversity of *Lactobacillus delbrueckii* strains, a technologically important lactic acid bacterium for the dairy industry. For this reason, *Lactobacillus delbrueckii* strains from the Saint-Nectaire Protected Designation of Origin (PDO) area were isolated and characterized. Genetic diversity was determined based on core genome phylogenetic reconstruction and pangenome analysis, while phenotypic assessments encompassed proteolysis and volatile compound production potential. A total of 15 *L. delbrueckii* ssp. *lactis* unique new strains were obtained. The genetic analysis and further proteolytic activities measurement revealed low variability among these Saint-Nectaire strains, while substantial genetic variability was observed within the *L. delbrueckii* ssp. *lactis* subspecies as a whole. The volatile compound profiles slightly differed among strains, and some strains produced volatile compounds that could be of particular interest for cheese flavor development. While the genetic diversity among Saint-Nectaire strains was relatively modest compared to overall subspecies diversity, their distinct characteristics and pronounced differentiation from publicly available genomes position them as promising candidates for developing autochthonous starter cultures for cheese production.

## 1. Introduction

*Lactobacillus delbrueckii*, a thermophilic lactic acid bacterium (LAB), occupies a significant place in the dairy fermentation industry. Among the six *Lactobacillus delbrueckii* subspecies, the two ssp. *bulgaricus* and ssp. *lactis* are widely used for their pivotal role in yogurt and cheese production, respectively. Despite its industrial importance, there is a lack of comprehensive studies addressing the genotypic and phenotypic diversity of *Lactobacillus delbrueckii*, especially its wild strains. Current research predominantly focuses on the subspecies *bulgaricus*, for which a rich diversity was reported in some studies but with strong environmental influences on strain distribution [1,2]. To date, few studies focus on ssp. *lactis*. Giraffa et al. [3] studied the phenotypic and genotypic diversity of *Lactobacillus delbrueckii* subsp. *lactis* starter strains, employing assays for acidifying and peptidase activities alongside RAPD-PCR and PFGE techniques. Their findings highlighted significant strain heterogeneity linked to isolation sources and periods.

In recent years, there has been a growing interest in developing autochthonous starter cultures for cheese manufacturing. It has been reported that such starters could preserve typical sensory properties of cheeses. Unlike commercial starters, these autochthonous cultures maintain the authentic flavors and aromas that define regional or traditional varieties, thereby safeguarding the identity and geographical indications of these cheeses [4,5,6]. Some studies also indicate that autochthonous starter cultures could exhibit favorable safety attributes, with the control of potentially harmful compounds production or their probiotic potential [7,8,9,10]. Developing new local microbial cultures also further contributes to sustainability and resilience of local dairy industries by strengthening food sovereignty and autonomy.

In this context, exploring intraspecies diversity appears to be an effective method for selecting strains with important technological characteristics and typicity signatures associated with a specific terroir [11]. The present study explores the genetic and functional diversity of wild strains of *Lactobacillus delbrueckii*. This diversity was explored in the Saint-Nectaire cheese-producing PDO area, one of the smallest PDO areas in Europe with only 1800 km^2^. The comparative analysis of the whole genome sequences of 15 unique new wild strains of *Lactobacillus delbrueckii* was carried out, and important functional properties of the strains were investigated, including proteolytic activities and production of volatiles compounds. The fifteen isolated *L. delbrueckii* ssp. *lactis* exhibited a strong genetic and technological homogeneity. However, the strains were distantly clustered from the public genomes, indicating a local typicity, associated with interesting functional and technological properties for their potential use as autochthonous starter cultures. To our knowledge, this work was the first to use the pangenome analysis to promote local *L. delbrueckii* subsp. *lactis* strains as starter culture. The use of microbial ecology principles in this study could offer cheese producers the benefits of autochthonous starter cultures.

## 2. Materials and Methods

### 2.1. Isolation and Identification of Wild Strains

Presumptive *Lactobacillus delbrueckii* strains were isolated from (i) 50 milk samples (heated for 6 h at 42 °C to enrich milk with thermophilic bacteria and promote the isolation of *Lactobacillus delbrueckii* strains) collected on 25 different farms in the Saint-Nectaire cheese-producing PDO area (France) in summer and in winter of the year 2020 and (ii) commercial starter cultures commonly used in Saint-Nectaire cheese production. Isolation was carried out on deMan, Rogosa, Sharpe (MRS) agar medium (Biokar Diagnostics, Beauvais, France) incubated at 42 °C for 48 h. Representative colonies were picked out of this medium, purified twice, and maintained frozen at −80 °C in an MRS broth medium containing 20% (*v*/*v*) glycerol. The Saint-Nectaire PDO area spreads over 69 municipalities between Puy-de-Dôme and Cantal (France), which are referenced in Table S1. The specifications for the PDO of Saint-Nectaire cheese describe the conditions for cow breeding, milk production, cheese manufacturing, and ripening of cheeses (https://www.inao.gouv.fr/show_texte/4840, accessed on 17 December 2023).

The species identification was carried out by amplification and sequencing of 16S rDNA gene (Eurofins Genomics, Konstantz, Germany) using WO2 and W18 as described previously [11,12]. To identify the partial 16S rDNA sequences obtained, a search of the NCBI GenBank DNA database was conducted using the BLAST algorithm. The percentage of similarity with DNA sequences deposited in this bank was determined. The 16S rDNA sequences were deposited in the NCBI GenBank database under accession numbers PP090971(H15BR1); PP090972 (73BR5); PP090973 (110BR2); PP090974 (110BR5); PP090975 (144BR1); PP090976 (149BR5); PP090977 (155BR2); PP090978 (H14BR1); PP090979 (H19BR1); PP090980 (46BR1); PP090981 (H29BR1); PP090982 (H28BR3); PP090983 (H24BR3); PP090984(H23BR3); PP090985(187BR1).

### 2.2. DNA Extraction and Sequencing

Genomic DNA was extracted from cell cultures with Nuclospin^®^ Tissue from Macherey Nagel (Düren, Germany) according to the manufacturer’s instructions. Final concentrations were measured with a Qubit™ fluorometer using the dsDNA Broad Range Kit (Thermo Fisher Scientific, Waltham, MA, USA). The extracted DNA was further sequenced using Illumina technology (Eurofins genomics, Konstantz, Germany). Library preparation and sequencing were handled by Eurofins genomics (Konstantz, Germany) using a Novaseq 6000 sequencing system (Illumina, San Diego, CA, USA). Sequencing reads from raw fastq files were filtered at Q30 with a minimal length of 110 bp with prinSeq [13]. Reads with remaining sequencing adapters were excluded with cutadapt V4.1 [14]. Each genome was assembled using Spades V3.13 [15] with the careful option and annotated with Prokka V1.5 [16]. The SN-strains genomes were compared to publicly available genomes. Accession numbers of the strains from this study and from the public genomes, as well as isolation sources, are referenced in Table 1.

### 2.3. Pangenome Assembly and Visualization

Annotated GFF3 files of the *Lactobacillus delbrueckii* subsp. *lactis* isolated from the Saint-Nectaire cheese-producing PDO area and public genomes were submitted to Roary [17] for pangenome analysis using default parameters. A gene presence–absence data matrix was derived and visualized using Phandango [18].

### 2.4. Phylogenetic Reconstruction

A core-genome single nuclear polymorphism (SNP) tree was created with Parsnp [19] on the Galaxy platform [20]. The resulting phylogenetic tree was visualized using iTOL (https://itol.embl.de/, accessed on 16 December 2023). Parsnp is a genome multi-alignment tool designed to align genome sequences. It aligns and provides the output as the multiple sequence alignment of given sequences, SNP variations, and the core genome phylogeny. To estimate the genome sequence similarities, the average nucleotide identity (ANI) was calculated using OrthoANI [21].

### 2.5. KEGG Functional Analysis

Kyoto Encyclopedia of Genes and Genomes (KEGG) numbers [22,23] for accessory genes were obtained using the eggnog-mapper v2 web tool [24]. Partial least squares discriminant analysis (sPLS-DA) was performed using the mixOmics package of R software V 4.2.3 (http://mixomics.org/, accessed on 14 November 2023).

### 2.6. Preparation of Model Cheese Curd

The model cheese curds (MCCs) were prepared according to Grizon et al. and Callon et al. [11,25]. In total, 40 mL of pasteurized milk (Ferme des Peupliers, Normandie, France) was incubated at 33 °C and inoculated with strains at 10^4^ cfu·mL^−1^. The milk was coagulated with 12 μL of calf rennet for 45 min at 33 °C and then centrifuged for 20 min at 20,000× *g* at 33 °C. The supernatant (lactoserum) was discarded, and the curd was incubated for 24 h in a temperature-controlled batch reactor programmed to simulate the decrease in temperature during the manufacture of Saint-Nectaire cheese type (decrease from 33 °C to 9 °C over 24 h). Each strain was tested three times.

### 2.7. Production of Volatile Compounds Using the DHS-TDU GC-MS Method

Three grams of each MCC sample were precisely weighed into a 20 mL vial, which was sealed with a septum-equipped screw cap and stored at −80 °C. The samples were allowed to stabilize at 4 °C overnight (approximately 16 h) before analysis. On the day of analysis, the samples were placed on a 10 °C DHS plate. For the dynamic headspace extraction (DHS), the samples were subjected to agitation and heating, followed by headspace purging with inert gas through needles. The extracted compounds were trapped and concentrated on a temperature-controlled Tenax polymer. Removal of water was achieved through dry purging with inert gas. The DHS operating conditions were as follows: incubation temperature 30 °C, incubation time 3 min, needle temperature 120 °C, trap extraction Tenax polymer, 30 °C, 300 mL He volume, 30 mL/min He flow, dry purge 30 °C, 300 mL He volume, 50 mL/min He flow. For the desorption (TDU)—injection (CIS), the molecules were thermally desorbed from the trap using inert gas sweep and were subsequently injected through a cooled injection system (CIS, PTV) to enable a discrimination-free transfer to the GC column. The TDU operating conditions were as follows: inert gas He, initial temperature 30 °C, ramped at 60 °C/min to 290 °C, held for 7 min, transfer temperature 300 °C. The IS operating conditions were as follows: inert gas He, initial temperature −100 °C, ramped at 12 °C/s to 270 °C, held for 5 min. For the gas chromatography (GC), the compounds were injected onto a polar capillary column (HP-Innowax, Agilent Waldbronn, Germany) and separated using a temperature program: 40 °C for 5 min, followed by a ramp of 40 °C/min to 155 °C, and then a ramp of 20 °C/min from 155 to 250 °C, with a subsequent maintenance at 250 °C for 5 min. Identification (MS): identification of compounds was based on retention time, and mass spectra were compared to the NIST 2017 Mass Spectral Library. Chromatographic peaks were integrated using total ion current (TIC) or extract ion chromatogram (EIC) for sample comparison, with direct TIC vs. EIC peak area comparisons being avoided. Peak areas of blank samples were subtracted from the assay samples. The peak areas of compounds for MCC samples inoculated with strains were determined at 24 h of fermentation by subtracting the results of MCCs made without inoculation of bacteria. Each strain was tested three times, and the results represent the average of the three replicates. Data were normalized, and a heatmap was created using heatmaply packages of R software to visualize data.

### 2.8. Proteolytic Activities

The extraction of water-soluble nitrogen from the MCC samples was carried out according to the method described by Myagkonosov et al. [26] with some modifications. Five grams of MCC samples were mixed with 5 mL of deionized water and homogenized with a stomacher for 4 min. The resulting mixture was transferred to a volumetric flask, and the volume was made up to 100 mL with deionized water. The mixture was kept at 40 °C for 1 h with continuous shaking. The samples were centrifuged at 3000× *g* for 30 min. After centrifugation, the samples were cooled to 4 °C, and the upper fat layer was removed. The supernatant was separated and filtered with a pore size of 0.45 μm. The resulting filtrate was mixed with deionized water at a ratio of 1:5. Next, 3 mL of OPA (o-phtalaldehyde) reagent prepared according to Church et al. [27] was added to 300 μL of the solution, and after 2 min, absorbance was measured at 340 nm with a 7200 spectrophotometer Jenway (Dutscher, France). The results have been expressed in mmol·L^−1^ of glycine based on a calibration curve. The proteolytic activity of strains was determined at 24 h of fermentation by subtracting the results at 0 h of fermentation. Twenty-four hours is the production time after addition of lactic starters and before removing the molds. Each strain was tested three times. Results are expressed as means of the three repetitions.

### 2.9. Statistical Analysis

Statistical analysis on volatile compounds production data was performed using the XLSTAT software V 2022.4.1 (Addinsoft, Paris, France). The results are reported as means of area units (AU × 10^4^) ± standard deviation (sd × 10^4^). The normality of the data was checked using a Shapiro–Wilk test (*p* < 0.05). The normal distribution was validated, and therefore an ANOVA and Tukey’s test were used to compare the area units obtained. Differences between the mean values were considered significant at *p* < 0.05.

Statistical analysis on proteolytic activities data was performed using the XLSTAT software (Addinsoft, Paris, France). The results are reported as means ± standard deviation. The normality of the data was checked using a Shapiro–Wilk test (*p* < 0.05). The test rejected the hypothesis Ha of normality when the value of *p* was ≤0.05. A large number of variables did not have a normal distribution, and therefore a non-parametric test (Kruskal–Wallis) and post hoc comparison (Dunn procedure) were used to compare the concentrations obtained. Differences between the mean values were considered significant at *p* < 0.05.

## 3. Results

### 3.1. Isolation and Identification of 15 Lactobacillus delbrueckii ssp. Lactis Wild Strains

In this study, strains belonging to the *Lactobacillus delbrueckii* species were isolated from the Saint-Nectaire cheese-producing PDO area (France). All strains were identified on the basis of 16S rDNA sequence alignment using the NCBI blast algorithm. Strains showing a percentage of similarity equal to or higher than 99% with *Lactobacillus delbrueckii* DNA sequences available in this database were considered to be *Lactobacillus delbrueckii*. In total, 33 isolates from 15 farms were identified as *Lactobacillus delbrueckii*. A total of 15 unique strains collected throughout 15 farms of the geographical producing area (1 isolate per farm), together with 1 commercial strain, were selected and characterized for their genetic and technological properties. Conditions of isolation and EBI accession numbers are referenced in Table 1. The genome sequencing and assembly-related information are shown in Table S2.

**Figure 1:**
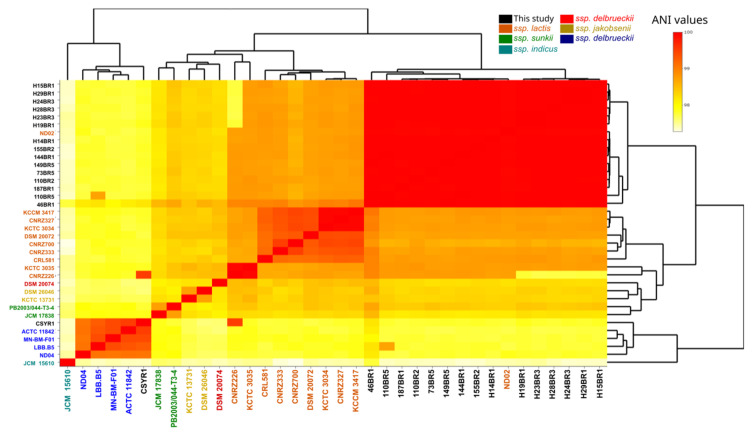
Average nucleotide identity (ANI), demonstrating the genomic distance among *L. delbrueckii* subspecies.

The calculation of average nucleotide identity (ANI) values could provide accurate taxonomic guidance based on whole-genome sequences. Baek et al. [28] demonstrated that ANI values among the *L. delbrueckii* subspecies were large enough to identify subspecies. Pairwise similarities for all SN-strains, commercial strains, and publicly available genomes were calculated using orthoANI [21] to identify subspecies to which SN-strains and the commercial strain belong (Figure 1). The results stated that all SN-strains formed a compact cluster, together with the ND02 genome. The ND02 strain was originally classified as *L. delbrueckii* subsp. *bulgaris*, but El Kafsi et al. [29] considered ND02 as representative of the subsp. *lactis.* They realized a multi-locus sequence typing analysis and identified ND02 as part of the ssp. *lactis* or ssp. *delbrueckii* cluster. Using a 16S rRNA alignment, ND02 shared nucleotides conserved in the ssp. *lactis* and *delbrueckii* strains that differ from the ssp. *bulgaricus*. They concluded from these results that ND02 was misclassified and belonged to the *lactis* subspecies. The results obtained here suggest that the strains belonged to the *L. delbrueckii* ssp. *lactis* subspecies. In contrast, the commercial strain CSYR1 belonged to *L. delbrueckii* ssp. *bulgaricus*. Since the study focused on wild strains of *L. delbrueckii*, genomic analysis did not include the CSYR1 strain to avoid bias in comparative analysis. Genomic analysis focused on *L. delbrueckii* ssp. *lactis* SN-strains and publicly available genomes.

### 3.2. Genetic Homogeneity among SN-Strains

#### 3.2.1. Pangenome Analysis

**Figure 2:**
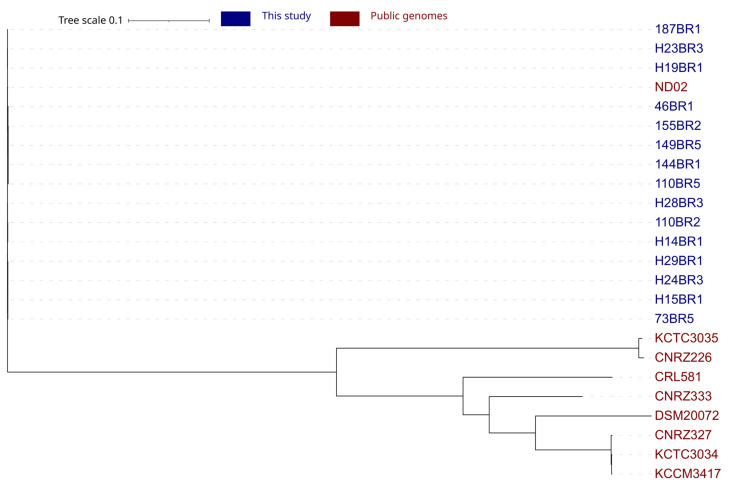
Phylogenetic trees of the 24 *Lactobacillus delbrueckii* strains based on single nucleotide polymorphisms.

**Figure 3:**
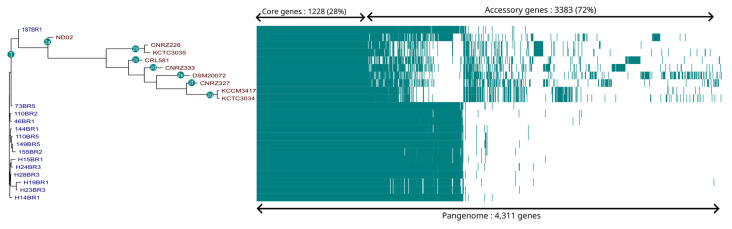
Clustering of strains associated with the visualization of the *Lactobacillus delbrueckii* pangenome. The pangenome was visualized based on the Phandandgo software (http://jameshadfield.github.io/phandango/#/, accessed on 17 November 2023; Hadfield et al., 2018 [18]). In the Roary matrix, genomes are shown as rows, and homologous gene clusters are depicted as columns. The presence of a gene cluster in a genome is indicated by blue. Core gene clusters found in all genomes are shown on the left side of the matrix. Strain names colored in blue were isolated in this study, and strain names colored in red were from public databases.

For exploring intraspecies diversity, a phylogenetic analysis based on core-genome single nucleotide polymorphisms (SNPs) was assessed (Figure 2). Interestingly, the first clade consisted of all Saint-Nectaire strains and the ND02 genome. The second clade included all other references genomes. The unique clade grouping all SN-strains revealed an absence of diversity within these strains. Phylogenetic analysis provides information about relationships between strains of a species using core genes. Such analysis, however, could not provide a comprehensive overview of the intraspecies genetic diversity, as it does not include the distribution of accessory genes across genomes. In contrast, pangenome analysis identifies ecological differences between genomes of a given species, determining the presence or the absence of all genes of a genome in a given strain. To evaluate the overall genetic diversity of *Lactobacillus delbrueckii* ssp. *lactis* isolated from a small geographical area, pangenome analysis of the 15 Saint-Nectaire strains and 9 publicly available genomes was performed by clustering the genes encoding complete protein sequences into core and accessory genomes using Roary (Figure 3) [17]. By definition, the core genome is the set of genes shared by at least 95% of the genomes and consists of genes that probably encode essential functions for the cell growth, while the accessory genome is shared by a subset of the genomes tested (less than 95% of the genomes tested) and encodes functions that confer selective advantages to a strain [30]. Considering this, from the 4311 genes constituting the pangenome of the *Lactobacillus delbrueckii* ssp. *lactis* genomes studied here, 1228 (28%) were core genes and 3083 (72%) were accessory genes. In a pangenome analysis of the whole *L. delbrueckii* species, Baek et al. [28] reported similar values, with a pangenome of 4332 genes, comprising 25% core genes and 75% accessory genes. The large proportion of accessory genes reported here suggested a high genetic diversity in these strains.

In total, pangenome analysis of the 23 *Lactobacillus delbrueckii* ssp. *lactis* strains highlighted two main clusters with differences in accessory genes content for each of them (Figure 3). The second cluster could be divided in seven sub-clusters, named 2a to 2g (Figure 3). The generated clustering based on the pangenome was concordant with the phylogenetic reconstruction based on SNPs in defining the relationships among strains, with the exception of ND02 that was part of the second cluster (Figure 3). Accordingly, the genetic diversity observed was not the consequence of the SN-strains genomes included, since they were clustered together.

A functional annotation with KEGG orthology was performed to understand the main differences between the accessory genes of the different clusters and to identify putative functional specificities of the SN-strains. The accessory genes positively annotated with a KEGG ko number were divided into 29 functional KEGG pathways, including 5 categories grouping almost 51% of these genes, which are “Protein families: signaling and cellular processes” (13% of the total annotated genes), “Carbohydrate metabolism” (12%), “Protein families: genetic information processing” (10%), “Membrane transport” (10%), and “Protein families: metabolism” (6%; Figure S1).

**Figure 4:**
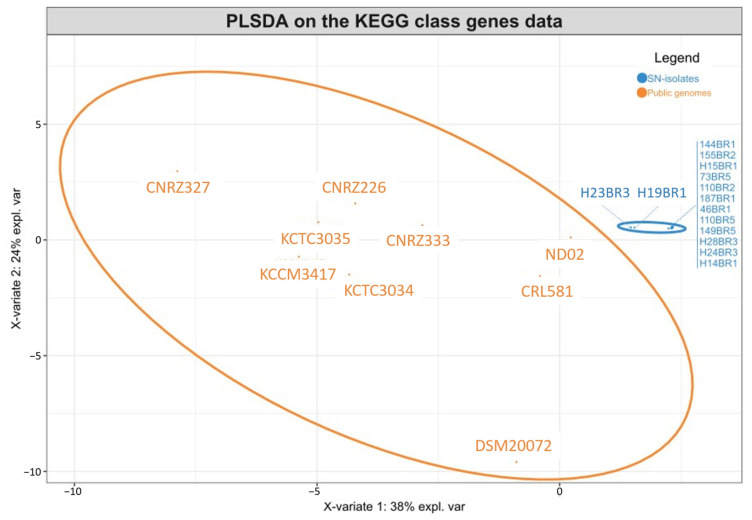
Sample plots from the PLS-DA analysis performed on accessory gene numbers of the KEGG class for the 8 groups.

**Figure 5:**
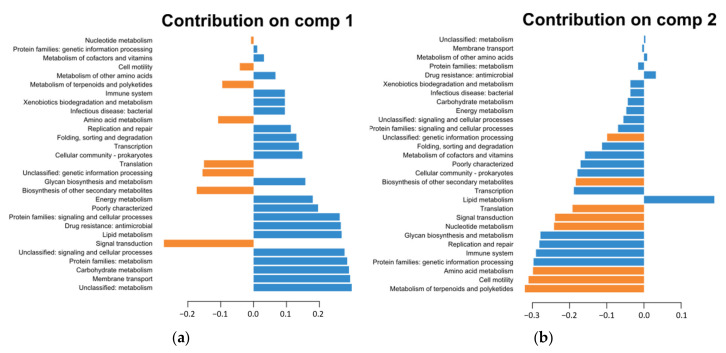
Plot loadings representing the variable’s contribution on component 1 (**a**) and on component 2 (**b**) of the PLS-DA analysis. Blue plots represent the SN-strains, while orange plots represent the public genomes.

The hierarchical clustering based on KEGG annotations reveals three groups similar to the pangenome clustering (Figure S1). A sparse variant of a partial least squares discriminant analysis (sPLS-DA) was performed (Figure 4) to identify KEGG pathways that differentiate strains into these groups. The results showed that the SN-strains are grouped together, while public genomes are more scattered, stating that no heterogeneity could be observed from the accessory genes of the SN-strains, while the diversity of the sub-species *lactis* as a whole seemed to be important. The SN-strains were separated from the public genomes predominantly along component 1, indicating that component 1 has a strong influence on the separation between the two groups (Figure 3). The bar plots for the contributions on component 1 showed which KEGG pathways are most discriminant for each component (Figure 4). Accordingly, the following KEGG pathways stand out as the most representative of the SN-strains: “Unclassified: metabolism”, “Membrane transport”, “Carbohydrate metabolism”, “Protein families: metabolism”, and “Lipid metabolism”. The second component mainly separated SN-isolates from the DSM20072 genome (Figure 4), and the main contributor of this separation was the “Lipid metabolism” KEGG pathway (Figure 5b). Carbohydrate metabolism is considered the most important metabolism pathway in lactic acid bacteria for their use as starters, as it directly affects the rate of milk acidification. Lipid metabolism is also an important biochemical process because lipolysis can contribute positively to cheese aroma or lead to a rancidity defect [31]. A high number of genes encoding for the oligopeptides transport system were identified in the « Membrane transport » functional categories for this cluster (Figure S2). Peptide transporters are key components of the proteolytic system in lactic acid bacteria, and proteolysis is one of the most important biochemical event during cheese production, with a major impact on flavor and texture [32,33,34]. In contrast, the following KEGG pathways stand out as among the most representative of the public genomes (Figure 5): «Unclassified: genetic information processing», «Protein families: genetic information processing», «Signal transduction». The abundant genes associated with these categories could increase the survival ability of strains when facing the change in environmental stress conditions. The manufacture of cheeses exposes starters to various environmental stresses (low pH, osmotic stress, and high pressure) [34], and such genes could therefore confer advantages in cheese production. Another important category, «Amino acid metabolism», that might affect the cheese-making process characterized this second cluster with a high number of genes implicated in aromatic amino acids biosynthesis (*aroE*, *aroA*, *aroK*, *aroD*, *aroQ*, *aroKB*) (Figure S3). Since aromatic amino acids are important precursors of flavor compounds, genes encoding enzymes of the biosynthetic pathways for these amino acids are very interesting in the context of cheese-making [35].

#### 3.2.2. Carbohydrate Putative Metabolism

**Figure 6:**
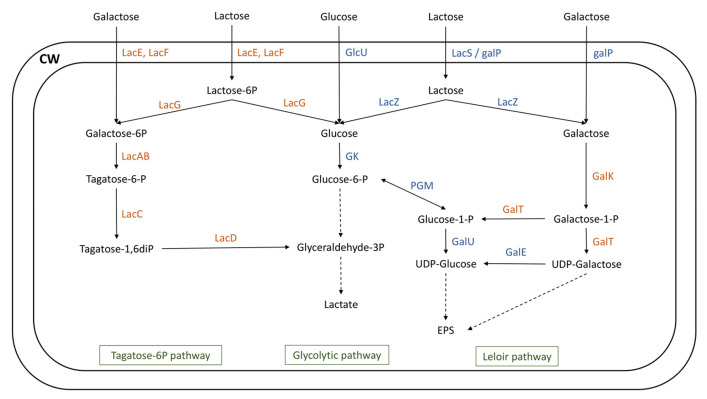
Carbohydrate transport and metabolism in the *Lactobacillus delbrueckii* genomes. Enzymes colored in blue are part of the core-genome, while those colored in brown are part of the accessory genome. GalE, phosphoglucomutase; GalK, galactokinase; galP, galactose and lactose permease; GalT, galactose 1-P uridylyltransferase; GalU, UDP-glucose pyrophosphorylase; GK, glucokinase; GlcU, glucose permease; LacAB, galactose-6-phosphate isomerase; LacC, tagatose-6-phosphate kinase; LacD, tagatose 1,6-diphosphate aldolase; LacE, PTS family lactose porter, EIICB components; LacF, PTS family lactose porter, EIIA components; LacG, 6-phospho-β-galactosidase; LacS, lactose permease; LacZ, β-galactosidase; PGM, phosphoglucomutase.

The capacity of lactic acid bacteria strains to metabolize carbohydrates holds significant importance in dairy fermentation, as it directly influences the rate of milk acidification. El Khafsi et al. [29] reported that *L. delbrueckii* ssp. *lactis* possessed the capacity for metabolizing a wide range of carbohydrates. Nonetheless, they also reported significant variability within the strains, showing differences in both number and types of metabolized carbohydrates among individual strains. The lactose, glucose, and galactose transport and utilization systems were analyzed in the genomes of the SN-strains and in the included genomes (Figure 6).

The fermentation processes carried out by LAB extensively rely on the utilization of lactose, a disaccharide consisting of two moieties, namely glucose and galactose [36]. Lactose utilization requires transport systems, encoded by a permease system (*galP* or *lacS*) or a phosphoenolpyruvate-phosphotransferase system (PEP-PTS) encoded by *lacEF*. After internalization into the cell, lactose is cleaved into galactose and glucose by a cytoplasmic β-galactosidase (*lacZ*), and galactose can be metabolized thanks to two pathways: Leloir or tagatose-6-phosphate (tagatose-6P) pathway [37]. The resulting glucose moiety is phosphorylated to glucose-6-P by glucokinase (*gk*) and is further utilized through the glycolytic pathway. LAB can also independently internalize glucose (thanks to the GlcU transporter) and galactose (using the *lacEF* PEP-PTS transporter or *galP* permease). Iskandar et al. [38] suggested that the transport system utilized for the internalization of galactose orientates the carbon flux towards one specific pathway. An internalization using a PEP-PTS system is thus linked to the tagatose-6P pathway, while the utilization of a permease system is linked to the Leloir pathway.

In the Leloir pathway, galactose is converted to glucose-1-phosphate by the galRKTEM gene cluster, which consists of the regulator GalR, galactokinase (GalK), galactose-1-phosphate uridylyltransferase (GalT), UDP-glucose-4-epimerase (GalE), and galactose mutarotase (GalM) [37,38].

In the tagatose-6P pathway, lactose-6P is hydrolyzed by a phospho-β-galactosidase (encoded by *lacG*) into glucose and galactose-6P. The latter is then transformed into glyceraldehyde-3P via a series of reactions encoded by *lacAB* (galactose-6-phosphate isomerase), *lacC* (tagatose-6-phosphate kinase), and *lacD* (tagatose-1,6-diphosphate aldolase) [37,39].

The 15 genomes of SN-strains investigated all harbored a PEP-PTS systems as well as specific permeases responsible for lactose, galactose, and glucose transport. Furthermore, genes responsible for their metabolism (Figure 6) were detected in these genomes (Table S3). On the contrary, none of the supplementary genomes included in this study possessed all the genes involved in the utilization of these carbohydrates, except in the *L. delbrueckii* ssp. *lactis* ND02 genome.

In *L. delbrueckii* ssp. *lactis* genome DSM20072, a gene encoding for galactokinase (galK) was not found. It was previously demonstrated that the deletion of this gene in the *Streptococcus mutans* UA159 strain significantly reduced its capacity to utilize lactose and galactose. Weiss et al. [40] reported a galactose-negative but a lactose-positive phenotype for the *L. delbrueckii* ssp. *lactis* DSM20072 strain. In *L. delbrueckii* ssp. *lactis* genomes CNRZ327 and CNRZ333, tagatose-6-phosphate kinase encoding by lacC was not found. However, Zeng et al. [41] demonstrated that the absence of this gene had a low impact on the utilization of lactose and galactose in the *S. mutans* UA159 strain. El Kafsi et al. [29] reported galactose-negative and galactose-positive phenotypes for the *L. delbrueckii* ssp. *lactis* CNRZ327 and CNRZ333 strains, respectively. The galactose- and lactose-negative phenotype of the CNRZ226 strain reported by El Kafsi et al. [29] signifies that this strain seemed to be unable to ferment lactose and galactose due to incomplete Leloir and tagatose-6P pathways (Figure 6). Although the phenotype of the KCTC3035 strain has not been reported in the literature, its profile, similar to the CNRZ226 strain (Figure 6), may indicate an identical fermentation phenotype.

El Kafsi et al. [29] studied the carbohydrate metabolism potential in the genomes of five *L. delbrueckii* ssp. *lactis* and highlighted a high level of variability within the subspecies. Our results corroborate their findings, with seven profiles of lactose, galactose, and glucose metabolism potential (Figure 6, Table S3). SN-strains all had a similar profile, reflecting a strong homogeneity. These results indicate that SN-strains appeared to be able to metabolize both glucose and galactose moieties of lactose, a key feature of dairy lactic acid bacteria. Although these analyses covered only three carbohydrates, they confirmed the KEGG annotations of accessory genes and revealed that SN-strains seemed to possess a higher number of genes implicated in carbohydrates metabolism in comparison with other genomes. However, experimental procedures are required to identify the ability of these strains to ferment these carbohydrates.

#### 3.2.3. Proteins and Peptides Putative Metabolism

The proteolytic system of LAB, and especially that of *L. delbrueckii* species, is essential for the supply of amino acids essential for their growth, as milk does not contain adequate concentrations of free amino acids [42]. This pathway is also of industrial importance since its derivates are known to contribute to the formation of texture and flavor of cheeses [32]. In *L. delbrueckii* ssp. *lactis* strains, proteolysis is initiated by a cell-envelope proteinase (CEP) encoded by *prtL,* which is responsible for the casein hydrolysis into oligopeptides [43]. The second step includes peptides transport into the cell by the Opp system [44], which are then degraded by intracellular peptidases into smaller peptides and amino acids [45].

The search for *prtL* genes was conducted in the genomes of the *L. delbrueckii* ssp. *lactis* genomes studies here. The SN-strains H19bR1 and H23bR3 and references genomes KCTC3035 and CNRZ226 did not appear to possess the specific cell-wall-bound proteinase. All other SN-strains and the reference genomes owned a single copy of the CEP gene *prtL* (Table S4). The presence of a cell-envelope proteinase in the genomes of LAB is a prerequisite to ensure proteolysis in milk, as it plays a critical role in the first step of caseins hydrolysis. As a consequence, strains without this proteinase should exhibit low proteolytic activities in fermented milk products [46].

The genome analysis revealed the presence of an oligopeptides ABC transporter encoded by the *opp* gene cluster in all SN-strains genomes and in some reference genomes. This peptides transport system was not found in the genomes of the *L. delbrueckii* ssp. *lactis* KCCM317, KCTC3034, CNRZ327, and DSM20072 strains. The *opp* transport system operon encodes two ATP-binding proteins (OppD and OppF), two membrane proteins (OppB and OppC), and a substrate-binding protein (OppA, Table S4), as previously reported by Brown et al. [47]. However, various peptides uptake systems were characterized in lactic acid bacteria but were not investigated in this study, such as the OPT oligopeptides ABC transporter system, encoded by the *optABCDF* operon, previously characterized in *L. delbrueckii* strains [47].

Additionally, a total of 13 peptidases were identified in the genomes studied, including *pepC*, *pepM*, *pepF*, *pepO*, *pepDA*, *pepV*, *pepT*, *pepP*, *pepQ*, *pepX*, *pepR*, and *pepI*, present in the core-genome and *pepN* present in the accessory genome, as it was absent in the genome of the *L. delbrueckii* ssp. *lactis* CNRZ327 strain. Elean et al. [48] analyzed the proteolytic system of 26 *Lactobacillus delbrueckii* strains, including 8 spp. *lactis* strains, and reported 15 peptidases that were part of the core-genome and 1 of the accessory genome (*pepL*).

Caseins hydrolysis potential seemed to be homogeneous among *L. delbrueckii* ssp. *lactis* genomes studied here, including within SN-strains, since similar profiles were observed in the proteolysis enzymatic pathway, except for some strains. However, most SN-strains harbored a complete pathway, which could indicate an interesting potential during the cheese-making process.

### 3.3. Phenotypic Analysis with Technological Interests

#### 3.3.1. Proteolytic Activities

**Figure 7:**
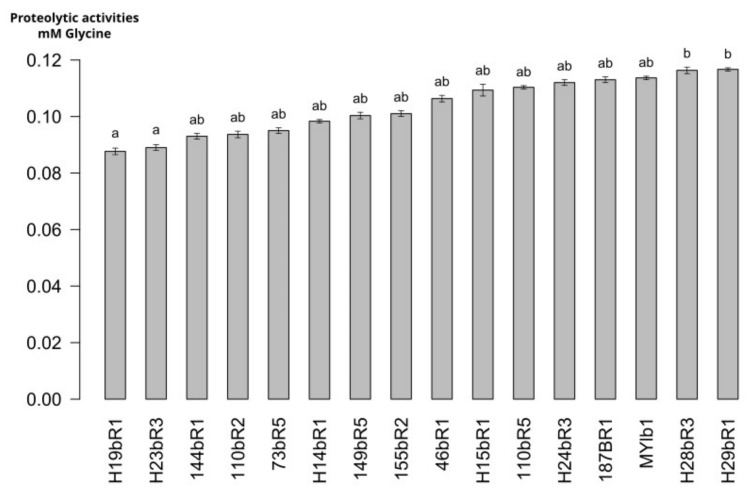
Proteolytic activities of *L. delbrueckii* ssp. *lactis* strains (a,b): mean values without a common superscript are significantly different (*p* < 0.05) according to the Dunn procedure test.

The proteolytic activities of the SN-strains and one commercial strain of *Lactobacillus delbrueckii* were evaluated in a model cheese curd at 24 h of fermentation, and the results are presented in Figure 7. The proteolytic activities varied slightly, from 0.088 to 0.117 mmoleq Glycine.L−1 for H19BR1 SN-strains and H29BR1 SN-strains, respectively. The proteolytic activities of the two SN-strains H19BR1 and H23BR3 were significantly different from the two SN-strains H28BR3 and H29BR1. The genomic analysis of the strains revealed that both SN-strains H19BR1 and H23BR3 did not harbor the specific CEP encoding by the *prtL* gene, which is essential to enable strains to hydrolyze milk caseins [47]. These two SN-strains showed the lowest activities among the tested strains. The proteolytic activities of the 11 other SN-strains were not significantly different (*p* < 0.05) from the two references strains and therefore exhibit interesting proteolysis potential for their use as starter culture. Proteolysis contributes significantly to cheese flavors by liberating peptides and free amino acids that undergo secondary reactions [32].

#### 3.3.2. Production of Volatile Compounds

Important enzymatic pathways and biochemical reactions induced by microbial communities lead to the formation of flavor volatile compounds that importantly contribute to flavor development of cheeses [49]. The production of volatile compounds by SN-strains and two commercial strains was investigated in model cheese curd using the DHS-TDU GC-MS technique. The strains were compared to a control commercial starter culture (CCSC) and an *L. delbrueckii* strain isolated from the CCSC starter (RCS).

The hierarchical clustering associated with the heatmap visualization (Figure S4) demonstrated that SN-strains, the control commercial starter culture, and the commercial strain could be divided in five groups according to their volatile flavor compound profiles. Strains H19BR1 and H23BR3 were grouped with the reference commercial strain RCS, suggesting that their profiles are similar to that of the reference strain. Strain 46BR1 formed a group with the reference starter culture CCSC. SN-strains 155BR2 and 144BR1 formed a group, and H15BR1 and 110BR2 were grouped together. All other SN-strains were grouped together. These results suggest slight variability within SN-strains.

In total, 40 volatile flavor compounds were identified, including 9 aldehydes, 14 ketones, 4 carboxylic acids, 9 alcohols, 3 esters, and 1 aromatic hydrocarbon (Table 2).

Carboxylic acid compounds are important components of dairy products [35,50]. Acetic acid, an important compound of fermentation associated with vinegar taste in some products [51,52], was detected in all MCC samples. Hexanoic acid, a volatile compound associated with cheesy, rancid, and sweat-like flavor in dairy products [53], was identified in MCC samples fermented by the CCSC and SN-strains 110bR5, 144bR1, H19bR1, and H23bR3.

Aldehydes have mainly been identified in dairy products. Hexanal and 3-methylbutanal were among the aldehydes most commonly detected in surface-ripened cheeses [50]. Hexanal is associated with green, lemon, and herbal notes [53,54,55,56], and its concentration in cheeses seems to be variable according to the ripening state. This compound was identified in 73BR5, 110BR2, H15BR1, H28BR3, H29BR1, and 187BR1 samples. 3-methylbutanal, a branched-chain aldehyde compound, was identified in CCSC and 73BR5 samples. This compound was previously detected in hard cheddar cheese and reported to be responsible for desirable flavor, and its associated notes have been described as “nutty”, “malty, cheese, green and dark chocolates” notes [50,57,58]. The volatile compound benzaldehyde, frequently detected in cheeses and associated with bitter almond and sweet cherry flavors [59,60], was detected in various samples (CCSC, 7BR5, 110BR2, H15BR1, H19BR1, H23BR3, H28BR3, H29BR1, 187BR1).

Alcohol compounds are the volatile compounds generally detected in highest numbers in surface-ripened cheeses [50]. Ethanol compound was detected in samples CCSC, 46BR1, 73BR5, 144BR1, 155BR2, H19BR1, and H23BR3. This compound contributes to dry, dust, and alcohol notes in cheeses [50]. 3-methylbutanol is often associated with fresh cheese, alcoholic, and floral [61]. This compound was identified in all the samples analyzed in this study. Helinck et al. [35] demonstrated that *L. delbrueckii* subsp. *lactis* was able to produce 3-methylbutanol compound from leucine by the action of an α-keto acid decarboxylase.

Ketone compounds are a key component of various dairy products [59,60,61]. Among all the ketones detected in this study, 2-pentanone (orange peel, sweet, and fruity notes) and acetoin were identified in all the samples. In contrast, 2,3-butanedione, an important volatile compound related to buttery flavor in dairy products [62,63], was only detected in CCSC, H19BR1, and H23BR3 MCC samples.

Some ester compounds positively contribute to the flavor in cheeses [64,65]. Notably, ethyl octanoate and ethyl hexanoate are important flavor compounds of cheeses due to their sweet, fruity, and floral notes [51]. They both were identified in H19BR1 and H23BR3 samples, and ethyl hexanoate was also identified in CCSC, 46BR1, and 144BR1 samples.

Toluene, an aromatic hydrocarbon frequently identified in cheeses [65,66,67,68,69,70] and associated with nutty and rancid odors, was detected in all samples.

Overall, minor differences were observed between MCC samples manufactured with the different strains, but important compounds were produced by SN-strains during the fermentation of MCC samples. It has been previously reported that using *Lactobacillus delbrueckii* subsp. *lactis* as a starter culture significantly affects the volatile compound profiles in hard cooked cheeses during ripening [69]. Volatile compounds identified in this study could positively affect the Saint-Nectaire cheeses aroma in the context of a utilization of SN-strains as starter cultures in this cheese type.

Liu et al. [69] studied the aroma of fermented milk produced by 28 *L. delbrueckii* subsp. *bulgaricus.* A sensory analysis indicated that fermented milks were classified in four different groups, including one “cheesy-type” and one “fermented-type”. A total of 95 volatile compounds in these two groups were identified by GC-IMS and GC–MS, and 12 aroma-active compounds were selected by GC-O-MS. Finally, six aroma-active compounds were determined as the key ones, including 2,3-butanedione, δ-decalactone, acet-aldehyde, butanoic acid, acetic acid, and hexanoic acid. Butanoic acid was identified as the decisive aroma compound for the cheesy-type, and hexanoic acid the decisive aroma compound of fermented-type. In our study, δ-decalactone and acetaldehyde were not identified in MCC samples fermented by the strains. However, compounds identified as 2,3 butanedione, butanoic acid, acetic acid, and hexanoic acid were found in MCC samples fermented by commercial starter CCSC, commercial strain RCS, and the two SN-strains H19BR1 and H23BR3. Consequently, the two latter strains seemed to be those that could most influence the “cheesy-type” or “fermented-type” sensorial characteristics of the MCC samples. However, sensorial analyses are required to validate this prediction.

## 4. Discussion

The use of commercial starters for the manufacture of artisanal cheeses is associated with a reduction in the variability of the cheese microbiota and thus a standardization of the final products [71]. Using the particular microbial richness and footprint of raw milk and cheese to develop indigenous starters is a real asset for ensuring the reproducibility of products without losing their typicality [72]. The isolation campaign allowed isolating 15 *L. delbrueckii* ssp. *lactis* strains from the Saint-Nectaire PDO area. The phylogenetic analysis, together with the pangenome generated with the 15 SN-strains, the commercial strain, and the 9 publicly available genomes, highlighted a high variability within the *Lactobacillus delbrueckii* ssp. *lactis* but a strong genetic heterogeneity among SN-strains. Tsuchihashi et al. [2] reported similar results in a study conducted on 226 *L. delbrueckii* strains isolated from raw milk in Hokkaido (Japan). They identified, in an MLSA analysis, a sub-cluster I-B1 accounting for 69.9% of isolated strains and reported that strains assigned to this cluster were dominant among *L. delbrueckii* isolated from raw milk in Hokkaido. The geographical origin and genomic evolution of strains appear to be closely linked in certain species. Song et al. [72] observed links between clusters identified using pan-core genomes analysis and geographical origins of *Lactobacillus delbrueckii* ssp. *bulgaricus* isolates. They explained those links with a possible evolution to adapt to their particular environments. The subspecies *L. delbrueckii* subsp. *lactis* has been studied less frequently. Nevertheless, Giraffa et al. [3] reported a correlation between clusters formed by random amplification of polymorphic DNA (RAPD-PCR) analysis and sources or periods of isolation of strains related to this subspecies. In this study, the separation of the *L. delbrueckii* spp. *lactis* SN-strains from the other strains, and the absence of diversity within SN-strains, indicated a strong influence of the Saint-Nectaire area geographical origin on the evolution of the SN-strains. It has been shown that local environments influence the composition of natural microflora [70,71,72,73,74,75]. Therefore, the environmental conditions of the small Saint-Nectaire PDO area and the Saint-Nectaire production process might be the reason for an adaptive evolution of the strains to the specific niche, leading to the observed low diversity.

The KEGG functional annotation of the accessory genes provided insights about the key functions of each cluster formed by the pangenome analysis. Interestingly, the accessory genes of the SN-strains were characterized by many KEGG functional categories that could be of serious interest as part of the development of indigenous starter culture adapted to cheese technology. The SN-strains seemed to own a high number of genes implicated in three key metabolisms in cheese production (carbohydrate, lipid metabolism, and proteolysis). The carbohydrate metabolism and proteolysis potential of the strains were investigated based on their whole genome analysis to validate these results. The lactose, galactose, and glucose metabolism potential from publicly available strains genomes included in this study seemed more reduced than that of SN-strains, except for the ND02 genome. El Kafsi et al. [29] and Song et al. [72] reported an association between fermentation profiles and in silico metabolic pathway analyses. Accordingly, fermentation profiles of SN-strains would be more varied than other genomes studied. However, experimental verification is required to identify the ability of these isolates to ferment these carbohydrates. Low variability of proteolytic potential was observed among *L. delbrueckii* ssp. *lactis* genome, although this is the only genetic characteristic which allowed us to differentiate some of the SN-strains. As only a search for specific genes was conducted, a more in-depth in silico analysis of the proteolytic systems would be interesting to assess diversity among strains. Elean et al. [48] conducted a study on the proteolytic system of 27 *L. delbrueckii*, including the search for specific genes implicated in proteolysis, a structural analysis of the CEPs, and an in silico analysis of the *prt* gene promoter; they concluded that *L. delbrueckii* ssp. *lactis* displayed a great variability. Though the genetic variability of the SN-strains was low in comparison with the diversity within the subspecies *L. delbrueckii* ssp. *lactis*, most of the strains exhibited genetic characteristics associated with metabolic potential of major importance for their use as starter culture in cheese production. To confirm these predictions, an assessment of technological properties was performed.

Exploration of the volatile compounds production appears to be an important parameter for the selection of autochthonous strains as candidates for the development of starter cultures. Randazzo et al. [74] studied the effect of wild strains on the volatile compounds of Pecorino Siciliano cheese. They demonstrated that the addition of wild strain had a significant impact on the typical flavor compounds of the cheeses. In this study, several profiles of volatile compounds were identified using the DHS-TDU GC-MS method, indicating that SN-strains showed a technological variability on this criterion. In total, 40 volatile compounds were identified, including aldehydes, alcohols, carboxylic acids, esters, and aromatic hydrocarbons compounds that were reported to contribute to fermented milk and cheese flavors in previous studies. These findings were consistent with those of Dan et al. [76], who compared 17 strains of *L. delbrueckii* ssp. *bulgaricus* to a control strain using the solid-phase microextraction (SPME) GC-MS method and reported variables profiles according to the strains. Furthermore, we identified two SN-strains, H19BR1 and H23BR3, and the SN-strain 46BR1 that exhibited a similar profile to the reference strain, and the reference commercial starter, respectively. However, the formers showed the lowest proteolytic activities. These strains could therefore be good candidates for the development of starter cultures for cheese manufacturing for their volatile compound production potential but should be used in association with other strains to ensure sufficient proteolytic activities. In this study, only the production of volatile compounds by SN strains in pure cultures was studied. However, Buchin et al. [68] suggested that *L. delbrueckii* spp. *lactis* could also affect the aroma of cheeses by providing precursors or nitrogen compounds that could favor other species. Although some major compounds were detected in MCC samples fermented with the different *L. delbrueckii* ssp. *lactis* strains, only a quantitative approach with the calculation of odor activity values could determine in a later stage if the compounds produced can contribute significantly to the aroma of cheeses.

We previously isolated and characterized a rich collection of *Streptococcus thermophilus* in terms of diversity from the Saint-Nectaire cheese PDO area [11], but the present study indicates that the possibility of isolating strains with high intraspecies genetic and functional variability from a small geographic area is not systematic. The absence of variability among *L. delbrueckii* SN-strains may be influenced by the isolation method used over the Saint-Nectaire PDO geographical area in the present study. The use of a single and somewhat selective medium (event if used classically in routine) could represent a bias in the isolation of the total available diversity. An interesting alternative strategy might have been to study the diversity upstream isolation, through a shotgun metagenomic sequencing approach, coupled with the pangenome analysis of metagenome-assembled genomes (MAGs). Such an approach would allow us to explore the possibility of isolating high genetic variability from a geographical area and to identify key functions to elaborate new strategies for the isolation of strains with technological interest. Zhai and Wei [76] constructed a genome collection of *Lactococcus lactis* by integrating MAGs and isolate genomes and assessed the genetic diversity of this species. They observed a pangenome in an open-state and highlighted an unexpectedly high diversity within the taxon. The method they used to understand the genetic and functional properties of *Lactococcus lactis* could be leveraged for the study of genetic diversity in a given geographical area with the aim to develop specific autochthonous starter cultures and should be tested for further diversity analysis.

According to the above results, the Saint-Nectaire cheese-producing area seems to be an important factor of adaptation of *Lactobacillus delbrueckii* subsp. *lactis*, leading to strong core- and pangenome homogeneity among strains. The adaptation of strains to this particular environment has led to a specific genomic footprint of SN-strains in comparison with genomes from other sources (public genomes), indicating a significant distinct specificity. In addition, the strains were able to produce important volatile compounds that could positively impact cheese aroma.

In conclusion, genetic and technological characterization of strains was an effective way to explore the opportunity to isolate candidates for the development of starter cultures in a limited geographical area. Moreover, in addition to the impact of these functions directly on the cheese-making process, studying the metabolic potential of each strain using these accessory genes’ functional annotation could provide clues for the production of starter cultures upstream of cheese manufacturing. Although low diversity was observed among SN-strains, their pronounced differentiation compared to publicly available genomes suggests a strong typicity of these strains, which, together with their technological characteristics, could make a significant benefit for their use as starter culture in Saint-Nectaire cheese production. The use of these autochthonous strains as starter cultures for producing Saint-Nectaire cheese could help to preserve the microbiological richness of raw milk from the area of production, maintaining the sensory properties of this cheese variety, and could significantly affect the local food autonomy. Further experiments are in process to pursue the development of new starter cultures including *L. delbrueckii* SN-strains from safety assessment to the technological validation in cheese-making large facilities.

## Figures and Tables

**Table 1:** *Lactobacillus delbrueckii* isolation sources with genomes assembly accession numbers analyzed in this study.

Strains	Isolation Source	Accession Number
H15BR1 (SN-strain)	Raw milk	GCA_963920275 ^1^
H29BR1 (SN-strain)	Raw milk	GCA_963920385 ^1^
H24BR3 (SN-strain)	Raw milk	GCA_963920445 ^1^
H28BR3 (SN-strain)	Raw milk	GCA_963920295 ^1^
H23BR3 (SN-strain)	Raw milk	GCA_963920345 ^1^
H19BR1 (SN-strain)	Raw milk	GCA_963920365 ^1^
H14BR1 (SN-strain)	Raw milk	GCA_963920425 ^1^
155BR2 (SN-strain)	Raw milk	GCA_963920285 ^1^
144BR1 (SN-strain)	Raw milk	GCA_963920375 ^1^
149BR5 (SN-strain)	Raw milk	GCA_963920315 ^1^
73BR5 (SN-strain)	Raw milk	GCA_963920405 ^1^
110BR2 (SN-strain)	Raw milk	GCA_963920335 ^1^
110BR5 (SN-strain)	Raw milk	GCA_963920435 ^1^
46BR1 (SN-strain)	Raw milk	GCA_963920395 ^1^
187BR1 (SN-strain)	Raw milk	GCA_963920355 ^1^
CSYR1 ^CS^	Commercial starter	-
ND02	Unknown	GCA_000182835.1 ^2^
CNRZ327	Environment	GCA_000751695.2 ^2^
KCTC3034	Sour milk	GCA_002016675.1 ^2^
DSM20072	Emmental cheese	GCA_002017855.1 ^2^
CNRZ700	Environment	GCA_000751275.1 ^2^
CNRZ333	Environment	GCA_000751235.1 ^2^
CRL581	Argentinian cheese	GCA_000409675.1 ^2^
KCTC 3035	Unknown	GCA_001888985.1 ^2^
CNRZ226	Environment	GCA_000751655.1 ^2^
DSM20074	Environment	GCA_001908495.1 ^2^
DSM 26046	Fermented beverage	GCA_001888925.1 ^2^
KCTC 13731	Environment	GCA_001888945.1 ^2^
PB2003/044-T3-4	Biological product	GCA_000179375.1 ^2^
JCM 17838	Fermented vegetable	GCA_001888965.1 ^2^
KCCM3417	Environment	GCA_001888905.1 ^2^
ACTC 11842	Bulgarian yogurt	GCA_000056065.1 ^2^
MN-BM-F01	Traditional fermented dairy	GCA_001469775.1 ^2^
LBB.B5	Home-made yogurt	GCA_001647065.1 ^2^
ND04	Fermented camel milk	GCA_002000885.1 ^2^
JCM 15610	Dairy fermented product	GCA_001908415.1 ^2^

^1^ EBI accession numbers sequenced in this study. ^2^ NCBI accession numbers of genomes publicly available. ^CS^ Commercial strain.

**Table 2:** Volatile compounds identified in MCC samples fermented with *Lactobacillus delbrueckii* SN-strains. Results are expressed as means of area units (AU × 10^4^) ± standard deviation (sd × 10^4^).

Strain	110BR2	110BR5	144BR1	155BR2	187BR1	46BR1	73BR5	CCSC	H15BR1	H19BR1	H23BR3	H28BR3	H29BR1	RCS
Esters														
Ethyl Acetate	-	-	-	-	-	274 ± 2.1 a	-	-	-	-	-	-	-	-
Ethyl hexanoate	-		497 ± 42.5 b	-	-	1425 ± 26.4 a	-	61 ± 0.7 c	-	30 ± 0.8 c	145 ± 3.2 c	-	-	133 ± 1.8 c
Ethyl Octanoate	-	-	-	-	-	-	-	-	-	11 ± 0.5 c	12 ± 0.3 b	-	-	13 ± 0.5 a
Ketones														
2-Butanone	2403 ± 239.2 b	-	-	-	-	-	-	2702 ± 38.9 a	2422 ± 272.1 ab	-	-	-	-	-
2,3-Butanedione	-	-	-	-	-	-	-	4499 ± 19.9 b	-	4703 ± 6.6 a	3290 ± 29.6 d	-	-	3745 ± 146 c
Methyl Isobutyl Ketone	314 ± 7.6 bcd	213 ± 15.5 gh	119 ± 13.3 i	421 ± 5.7 a	316 ± 2.9 bc	275 ± 3.8 def	343 ± 42.3 b	291 ± 6.3 cde	414 ± 10.4 a	273 ± 1.6 ef	317 ± 1.1 bc	244 ± 9.1 fg	191 ± 0.9 h	444 ± 4.3 a
Acetoin	31 ± 0.3 g	15 ± 0.2 g	54 ± 0.6 g	370 ± 8.4 f	42 ± 0.9 g	12447 ± 72.1 b	63 ± 0.0 g	13206 ± 81.2 a	85 ± 0.2 g	9327 ± 84.1 d	5930 ± 77.1 e	39 ± 0.2 g	23 ± 0.7 g	9928 ± 29.6 c
2,3-Pentanedione	-	-	-	-	25 ± 0.5 d	-	9 ± 0.2 e	123 ± 3.6 b	11 ± 0.2	120 ± 6.8 bc	115 ± 0.2 c	-	-	143 ± 2.1 a
2-Heptanone	-	164 ± 4.9 a	72 ± 0.4 c	-	-	23 ± 1.0 g	-	154 ± 5.3 b	55 ± 0.3 de	52 ± 0.2 e	59 ± 0.7 d	42 ± 0.1 f	46 ± 0.7 f	70 ± 0.2 c
Acetoin acetate	-	-	-	-	-	-	-	62 ± 0.3 d	-	652 ± 2.8 a	395 ± 3.6 c	-	-	464 ± 2.5 b
2-Hydroxy-3-pentanone	-	-	-	-	-	-	-	181 ± 5.6 c	-	149 ± 2.2 d	208 ± 5.6 b	-	-	236 ± 3.0 a
Acetophenone	-	-	-	-	-	-	-	12 ± 2.2 a	-	-	-	-	-	-
2-Propanone, 1-hydroxy-	-	-	-	-	-	-	-	55 ± 0.5 a	-	54 ± 0.5 b	-	-	-	43 ± 0.1 c
Acetone	1136 ± 47.3 f	705 ± 5.9 j	1330 ± 41.4 e	5848 ± 37.3 a	1077 ± 82.3 fg	1782 ± 55.1 d	979 ± 4.3 ghi	1130 ± 13.8 f	906 ± 4.5 i	3842 ± 6.1 c	5776 ± 99.2 a	1045 ± 6.6 fgh	919 ± 9.3 hi	4405 ± 57 b
2-Pentanone	684 ± 3 g	1205 ± 2.7 a	760 ± 11.4 f	803 ± 3.9 e	743 ± 19.4 f	983 ± 4.5 b	665 ± 1.6 g	748 ± 6.8 f	933 ± 5.2 c	542 ± 2.0 i	622 ± 3.1 h	964 ± 3.9 b	874 ± 5.0 d	390 ± 6.4 j
2-Nonanone	-	-	-	-	-	23 ± 0.5 a	-	14 ± 0.8 b	-	-	-	-	-	-
2,3-Dimethylhydroquinone	-	-	-	-	-	-	-	46 ± 0.9 a	-	-	-	-	-	-
Aldehydes														
2-Methylbutanal	-	-	-	-	-	-	146 ± 0.9 b	225 ± 14 a	-	-	-	-	-	-
3-Methylbutanal	-	-	-	-	-	-	1374 ± 90.7 a	1167 ± 25.5 b	-	-	-	-	-	-
Decanal	16 ± 0.3 a	-	16 ± 0.6 a	13 ± 1.0 b	-	-	12 ± 0.8 b	-	-	-	-	-	12 ± 0.5 b	-
Benzaldehyde	26 ± 0.3 e	-	-	-	118 ± 1.3 a	-	45 ± 0.9 b	8 ± 0.1 g	25 ± 0.4 e	11 ± 0.2 f	8 ± 0.0 g	43 ± 1 c	35 ± 0.2 d	-
Benzaldehyde, 4-methyl-	15 ± 0.5 c	-	-	-	-	-	35 ± 0.4 b	-	-	-	-	39 ± 0.3 a	-	-
Hexanal	66 ± 0.5 d	-	-	-	143 ± 8.8 a	-	42 ± 0.2 f	-	50 ± 0.8 e	-	-	97 ± 0.4 b	87 ± 0.9 c	-
Heptanal	34 ± 2.3 b	-	-	-	135 ± 14 a	-	-	-	-	-	-	-	-	-
Octanal	-	-	-	-	8 ± 0.2 a	-	-	-	-	-	-	-	-	-
2-Nonenal, (E)-	-	-	-	-	7 ± 0.2 a	-	-	-	-	-	-	-	-	-
Alcohols														
2-Butanol, (R)-	-	644 ± 2.7 a	-	-	-	-	-	-	-	-	-	-	-	-
1-Propanol, 2-methyl-	286 ± 2.4 j	423 ± 2.2 g	506 ± 3.9 f	416 ± 2.4 g	416 ± 4.8 g	559 ± 7 e	308 ± 4.7 i	938 ± 7.5 a	298 ± 1.5 ij	778 ± 5.7 b	761 ± 5.3 c	334 ± 2.7 h	251 ± 4.4 k	675 ± 3.8 d
3-Methyl-Butanol	36 ± 0.3 e	63 ± 0.2 e	34 ± 0.7 e	75 ± 0.2 de	37 ± 0.2 e	2966 ± 53.1 b	28 ± 0.0 e	4689 ± 100.8 a	60 ± 0.2 e	369 ± 5.1 c	115 ± 1.1 de	33 ± 0.4 e	28 ± 1.0 e	155 ± 3.6 d
3-Pentanol	-	-	-	-	-	-	-	509 ± 1.4 a	-	307 ± 7.4 b	156 ± 4.2 d	-	-	259 ± 5.2 c
Ethanol	-	-	457 ± 0.1 c	1443 ± 28.3 a	-	138 ± 6.6 de	211 ± 0.7 d	114 ± 2.6 e	-	110 ± 5.2 e	1356 ± 89.1 b	-	-	1336 ± 12.3 b
1-Butanol	153 ± 1.6 b	-	-	106 ± 0.2 e	168 ± 1.0 a	61 ± 0.6 g	147 ± 1.8 c	65 ± 0.1 f	124 ± 3.4 d	-	-	63 ± 0.1 fg	13 ± 0.3 h	-
1-Penten-3-ol	8 ± 0.3 b	-	-	9 ± 0.1 b	32 ± 1.3 a	-	6 ± 0.1 c	-	-	-	-	-	-	-
1-Pentanol	-	-	-	60 ± 0.1 b	183 ± 17.8 a	-	-	-	-	-	-	-	-	-
1-Octadecanol	-	-	-	-	-	-	-	48 ± 0.4 a	-	40 ± 0.2 b	-	-	-	-
Aromatic hydrocarbons														
Toluene	254 ± 1.7 b	186 ± 1.7 ef	155 ± 0.5 gh	188 ± 0.9 e	167 ± 1.3 fg	153 ± 1.8 gh	246 ± 13.7 b	222 ± 3.9 cd	226 ± 14.4 c	274 ± 1.7 a	144 ± 3.4 hi	203 ± 11.6 de	132 ± 2.1 i	157 ± 0.7 gh
Carboxylic acids														
Acetic acid	27 ± 0.3 ij	47 ± 0.6 gh	17 ± 0.6 jk	92 ± 0.2 e	57 ± 0.2 g	334 ± 3.2 c	10 ± 7.2 k	1143 ± 5.8 a	37 ± 0.8 hi	326 ± 1.5 c	241 ± 0.8 d	70 ± 1.0 f	41 ± 0.3 h	381 ± 11 b
Hexanoic acid	-	34 ± 0.3 b	16 ± 0.4 d	-	-	-	-	253 ± 4.4 a	-	23 ± 0.3 c	35 ± 0.3 b	-	-	36 ± 0.2 b
Octanoic acid	-	-	-	-	-	-	-	34 ± 1.9 a	-	-	-	-	-	-
Butanoic acid	-	165 ± 3.3 c	76 ± 0.2 d	-	-	-	-	1442 ± 24.2 a	-	1357 ± 2.7 b	-	-	-	180 ± 8.4 c

(a–k): mean values without a common superscript in each rows are significantly different (*p* < 0.05) according to the Tukey’s test.

## Data Availability

The data presented in this study are deposited in online repositories. Genomes can be found in the EBI repository under the project accession number PRJEB61322.

## Supplementary Materials

The following supporting information can be downloaded at: https://www.mdpi.com/article/10.3390/microorganisms12030512/s1, Table S1: Municipalities comprising the Saint-Nectaire cheese PDO area; Table S2: Genome sequencing and assembly-related information; Table S3: The lactose, glucose, and galactose transport and utilization systems; Table S4: The proteolytic systems of the strains; Table S5: Growth characteristics of the *Lactobacillus delbrueckii* SN-strains in MCC samples; Figure S1: Hierarchical clustering of strains based on the gene content summarized by KEGG pathways; Figure S2: Heatmap representing the lowest level of the KEGG pathway database (gene ko level) for the Membrane transport KEGG pathway; Figure S3: Heatmap representing the lowest level of the KEGG pathway database (gene ko level) for the amino acid metabolism KEGG pathway; Figure S4: Hierarchical clustering associated with heatmap of volatile metabolite profiles from MCC samples fermented by different *L. delbrueckii* ssp. *lactis* strains. Each square in the heatmap expresses normalized volatile peak area (means of three replicates) respective to the color range.
