# Supplementary material for: Genomic Characterization of Wild Lactobacillus delbrueckii Strains Reveals Low Diversity but Strong Typicity
Source: Microorganisms. 2024 Mar 2;12(3):512. doi: 10.3390/microorganisms12030512 (PMC10974765; doi:10.3390/microorganisms12030512)
Supplement: Supplementary file 1 [file microorganisms-12-00512-s001.zip › Table S1.pdf]

Table S1: Municipalities comprised in the Saint-Nectaire cheese PDO area

Cantal department				
Allanche	Beaulieu	Champs-sur-Tarentaine-Marchal	Chanterelle	Condat
Landeyrat	Lanobre	Lugarde	Marcenat	Marchastel
Montboudif	Montgreleix	Pradiers	Saint-Amandin	Saint-Bonnet-de-Condat
Saint-Saturnin	Ségur-les-Villas	Trémouille	Vernols	
Puy-de-Dôme department				
Anzat-le-Luguet	Ardes	Aurières	Aydat	Bagnols
Besse-et-Saint-Anastaise	Chambon-sur-Lac	Chassagne	Chastreix	Compains
Courgoul	Cournols	Creste	Cros	Dauzat-sur-Vodable
Egliseneuve-d’Entraigues	Espinchal	Grandeyrolles	LaBourboule	La Chapelle-Marcousse
La Godivelle	La Tour-d’Auvergne	Labessette	Larodde	Le Vernet-Sainte-Marguerite
Mazoires	Montaigut-le-Blanc	Mont-Dore	Murat-le-Quaire	Murol
Olloix	Orcival	Picherande	Rentières	Roche-Charles-la-Mayrand
Saint-Alyre-ès-Montagne	Saint-Diéry	Saint-Donat	Saint-Genès-Champespe	Saint-Nectaire
Saint-Pierre-Colamine	Saint-Victor-la-Rivière			
