# Supplementary figures and images for: Genomic Characterization of Wild Lactobacillus delbrueckii Strains Reveals Low Diversity but Strong Typicity
Source: Microorganisms. 2024 Mar 2;12(3):512. doi: 10.3390/microorganisms12030512 (PMC10974765; doi:10.3390/microorganisms12030512)

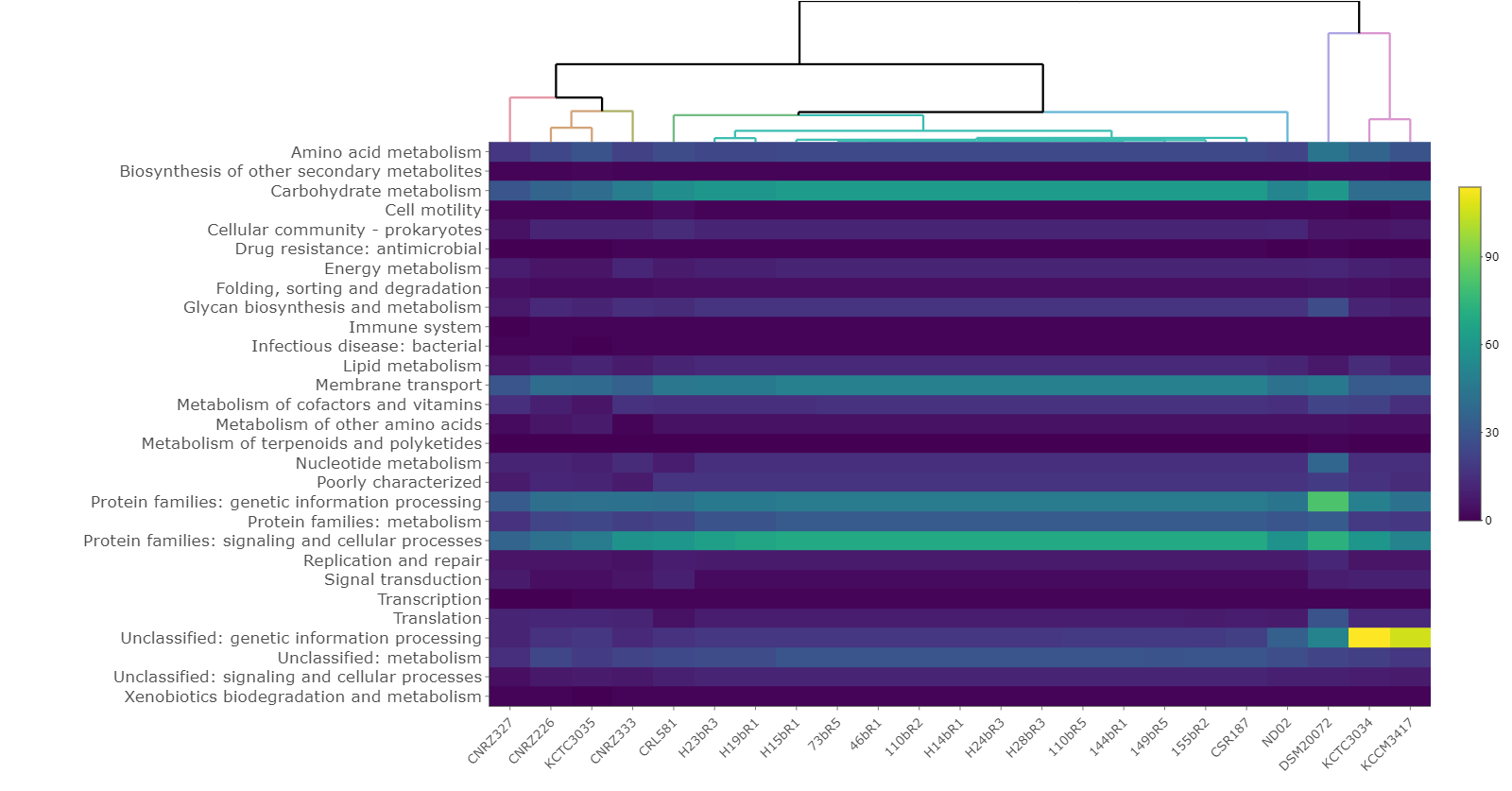

Supplement: Supplementary file 1 [file microorganisms-12-00512-s001.zip › Figure S1.png]

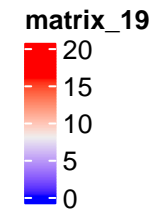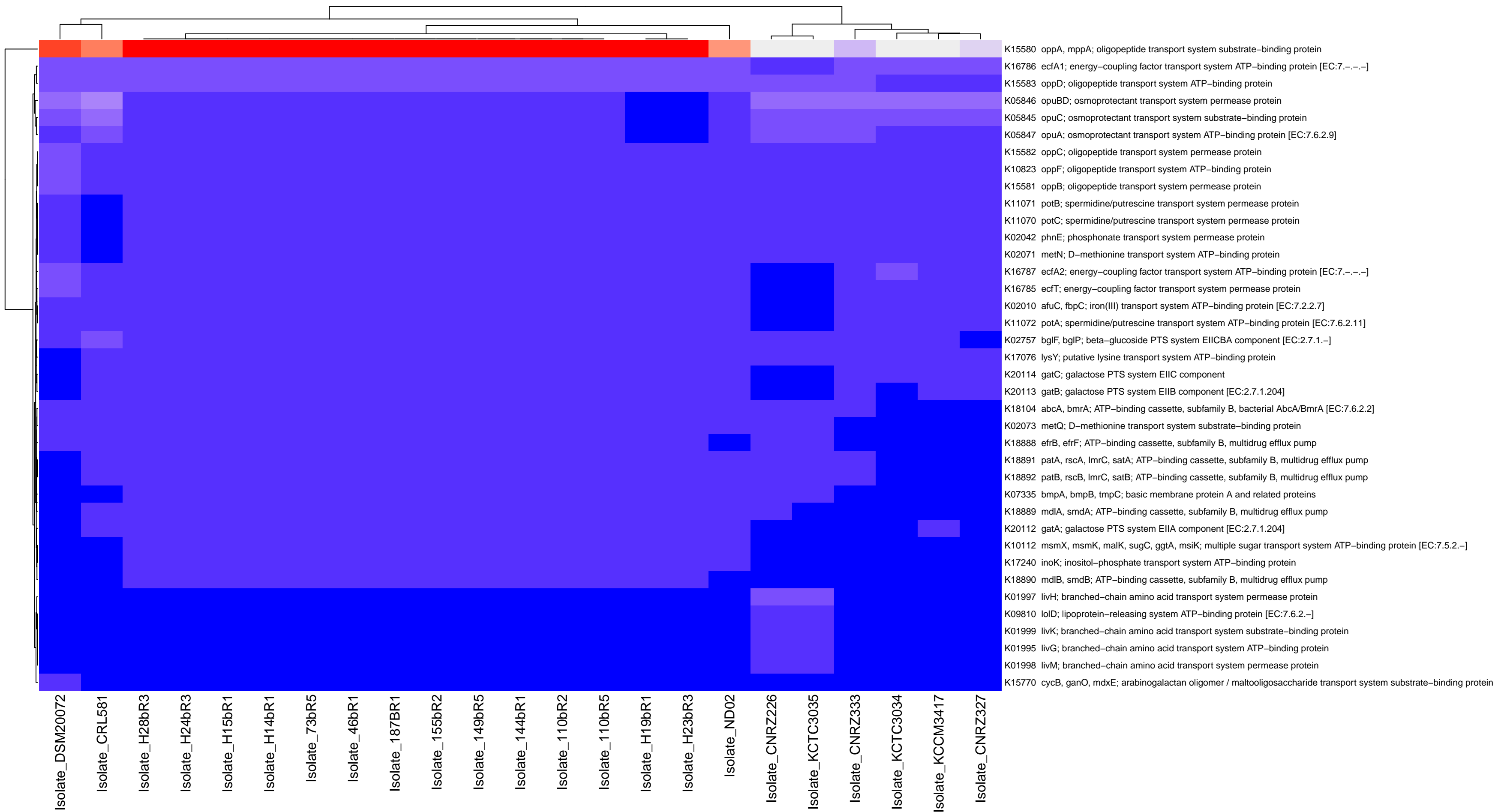

Supplement: Supplementary file 1 [file microorganisms-12-00512-s001.zip › Figure S2.pdf]

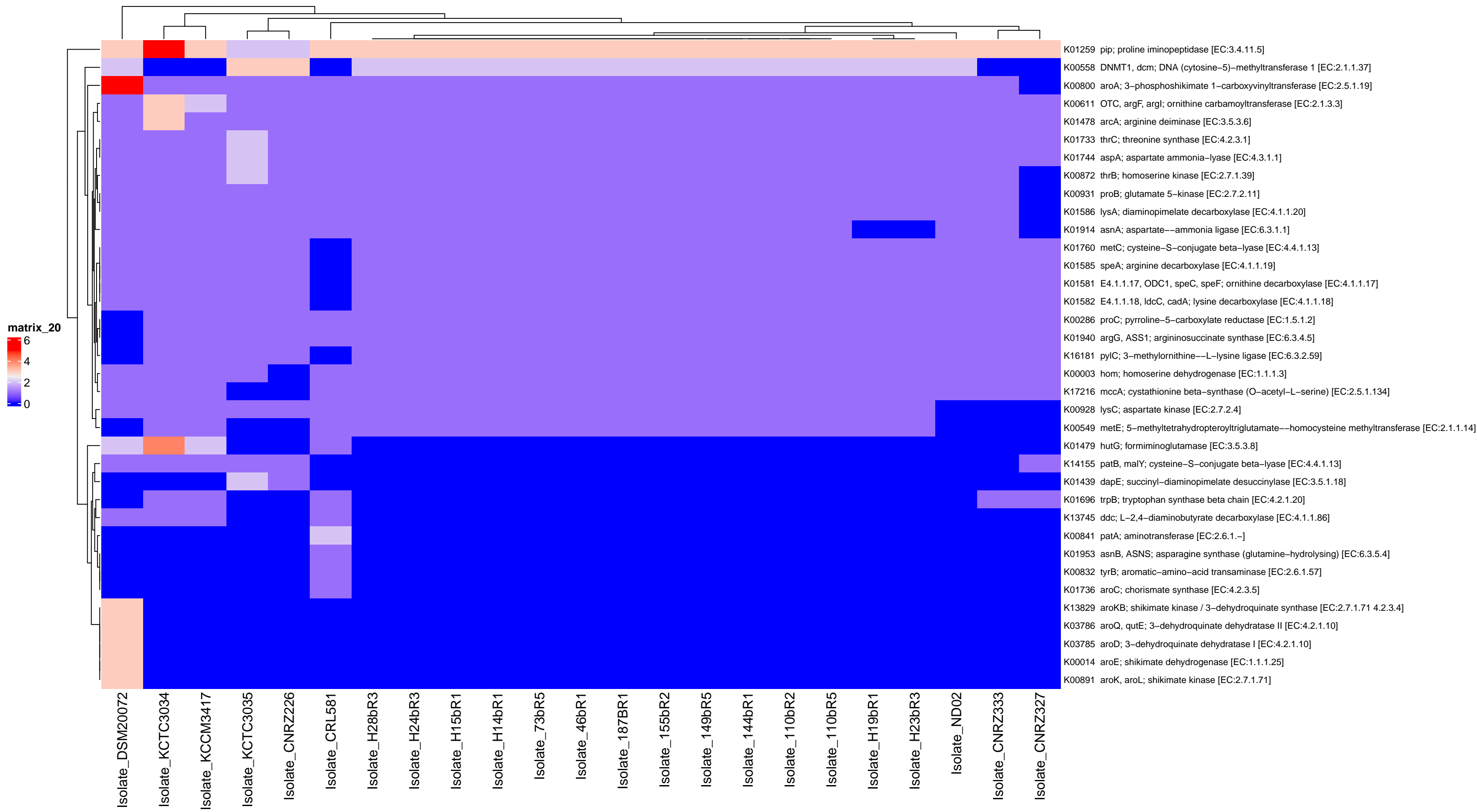

Supplement: Supplementary file 1 [file microorganisms-12-00512-s001.zip › Figure S3.pdf]

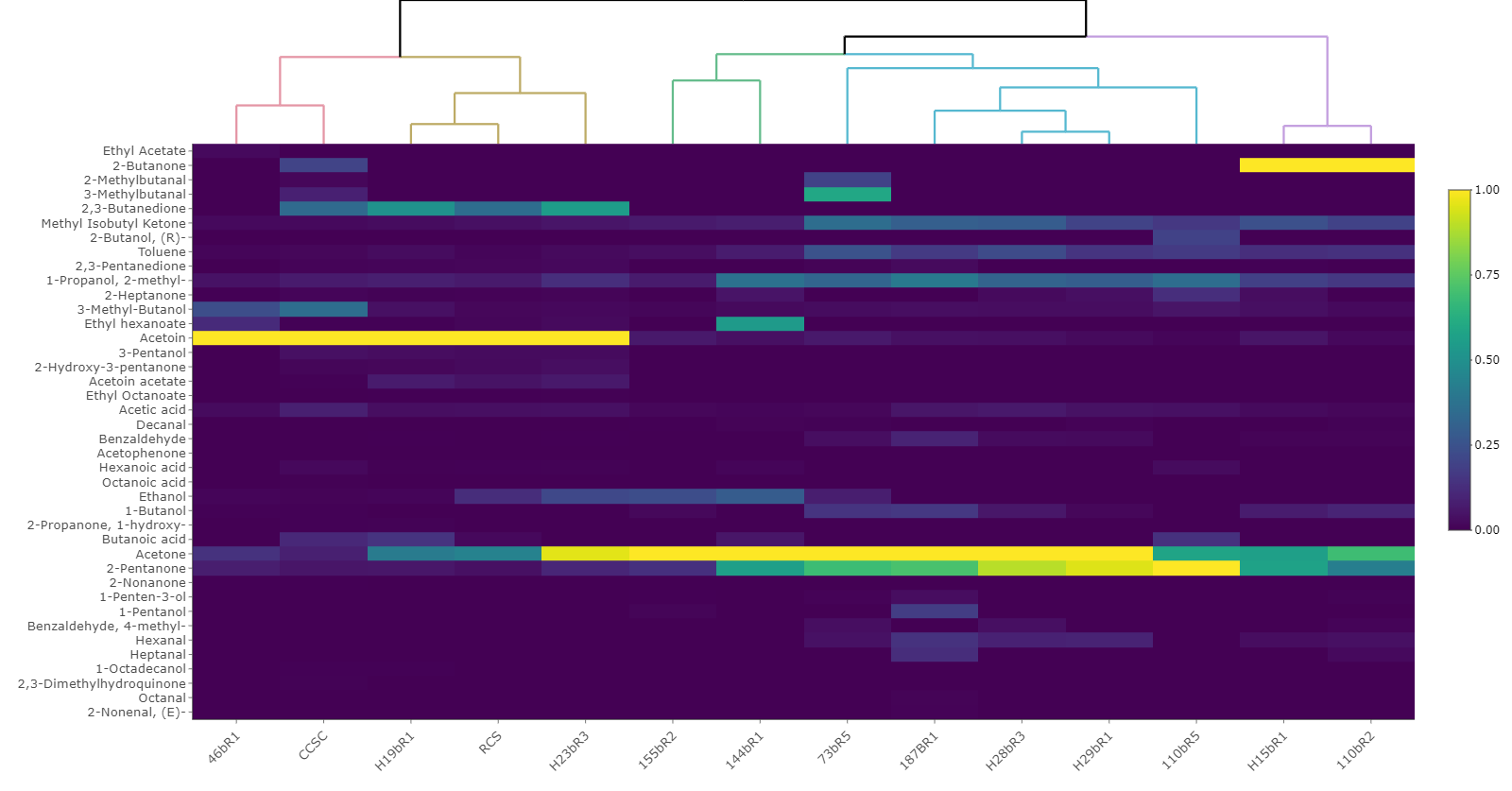

Supplement: Supplementary file 1 [file microorganisms-12-00512-s001.zip › Figure S4.png]
